# The Future of Sleeve Gastrectomy

**DOI:** 10.17925/EE.2016.12.01.37

**Published:** 2016-03-15

**Authors:** Michel Gagner

**Affiliations:** Herbert Wertheim School of Medicine, Florida International University, Miami, US; Hôpital du Sacre Coeur, Montreal, Canada

**Keywords:** Sleeve gastrectomy, bariatric surgery, obesity, metabolic surgery, type 2 diabetes

## Abstract

The International Diabetes Federation has recently published a position statement on bariatric surgery. Facing a global diabetes crisis, it has added various types of surgery on the gastrointestinal tract as powerful options among the armamentarium to normalise glycaemia, avoid regular medications and decrease costs in severely obese patients. Laparoscopic sleeve gastrectomy has been shown to be effective for the treatment of type 2 diabetes, with extremely low mortality and acceptable morbidity. It decreases caloric consumption, modifies gut hormones, changes gastric emptying, and lowers glycaemia. In the long term, if recurrences in weight or diabetes occur, it can be converted to a duodenal switch, potentially reaching 90% of patients with partial or complete remission.

Laparoscopic sleeve gastrectomy was initially described as a stage procedure for high-risk patients, followed six months later with either a full duodenal switch or gastric bypass.^1^ Laparoscopic sleeve gastrectomy is now the dominant weight-loss surgery in the US, an achievement that was never attained with the less invasive adjustable gastric banding. American patients are choosing this procedure at a ratio of 2:1, instead of a Roux-en-Y gastric bypass, which was the gold standard for several decades. Compared with gastric bypass, the procedure causes less short- and long-term morbidity; preserves the full length of the intestine for micronutrients absorption; preserves the pylorus for gastric emptying; causes less rebound hypoglycaemia; and has similar effects and rates of comorbidity resolution and percentage of excess weight loss.

From the surgeon’s perspective, the procedure is easier to perform (no intestinal anastomosis), with shorter anaesthesia and simplified follow-up protocols. In a recent study presented at the last annual meeting of the American Society for Metabolic and Bariatric Surgery (ASMBS) using the American College of Surgeons National Surgical Quality Improvement Program (NSQIP) database, Cleveland Clinic authors identified 72,000 patients with a body mass index (BMI) of at least 35 kg/m^2^ who had bariatric surgery between 2010 and 2013. In 2010, sleeve gastrectomy accounted for 9% of all weight loss procedures, while Roux-en-Y gastric bypass made up 58% and adjustable gastric banding 29%. By 2013, 49% of the procedures were sleeve gastrectomy and just 6% were gastric band procedures. Numbers have continued to grow, such that in 2014, sleeve gastrectomy rose to above 50% and is projected to be near 60% in 2015. In certain states like Michigan, that procedure has already reached 67% in 2014.

Like any surgical procedure, there have been, relatively few, complications, including potential gastric staple line leaks or bleeding (1–2%), occasional strictures (0.5%), and *de novo* gastroesophageal reflux disease GERD (5%). Some micronutrient deficiencies may occur and multivitamins should be given routinely, especially vitamin B12.

Several surgeons with more traditional views continue to perform gastric bypasses, especially when type 2 diabetes is involved, as it is subjectively believed that this operation has more durable effect on type 2 diabetes than sleeve gastrectomy. However, there has been documentation of recurrence after five to 15 years and *de novo* occurrences of type 2 diabetes in 25–40% of patients who are operated on. Hence, the complete effect of gastric bypass is substantially diminished over time, and is closer to 40–45%. Once a patient has a gastric bypass, it is very difficult to improve or revise this operation, to augment its effect. However, it is believed by another group of surgeons that sleeve gastrectomy is the proper ‘first’ procedure to do, because of its universality and easiness to transform to duodenal switch or other types of intestinal malabsorption surgery to counter weight regain and type 2 diabetes in the long term.^2^ Duodenal switch comprises a sleeve gastrectomy with a lower intestinal bypass, hence, when utilised as a second stage, the sleeve is left intact and the distal bypass added below; a fairly straightforward procedure. One has to review the dramatic effect of duodenal switches on diabetes to understand that it has a major role to play in type 2 diabetes, including insulin-dependent patients. Three longterm studies (Cornell University, New York University and a Belgian hospital) were presented at the last ASMBS meeting in November 2015. The Cornell group had demonstrated in a cohort of 190 patients a reduction from an overall rate of 58% of type 2 diabetes to zero (0%) after nine years, and also a reduction to zero (0%) with a haemoglobin A_1C_ above 5.6% after the same period. Dyslipidemia decreased from 45% to 2%.^3^ This corroborates Dr Picard Marceau’s remarkable study from Laval University in Quebec City, where type 2 diabetes was ‘cured’ in 93% of patients, 20 years after a duodenal switch.^4^ The procedure has been simplified lately with a single anastomosis duodenal switch or single anastomosis duodeno-ileostomy (SADI), with similar results after five years, but with lesser operating time, complications and diminished side effects, from a group in Madrid (Prof. Antonio Torres and Dr Andres Sanchez-Pernaute, from Hospital Clinico San Carlos). ^5^

These effects are not surprising when one notes that sleeve gastrectomy has a restrictive component due to its reduced volume (>80%), decreased total acid production, hormonal changes with reduced serum ghrelin production (70%), increased post-prandial glucagon-like peptide-1 and peptide YY 3–36, and is lately associated with increased circulating bile acids, changes to gut microbial flora and farsenoid-X receptor signalling.^6^ Hence, I believe the future of sleeve gastrectomy is bright and it will probably become the primary procedure in >75% of patients seeking weight-loss surgery and/or type 2 diabetes surgery.
